# Prevalence and Predictors of Chronic Kidney Disease in a Semiurban Community in Lagos

**DOI:** 10.1155/2019/1625837

**Published:** 2019-05-02

**Authors:** Innocent Ijezie Chukwuonye, Kenneth Arinze Ohagwu, Olufemi Oladipo Adelowo, Abali Chuku, Emmanuel Chukwuebuka Obi, Uwa Onwuchekwa, Ernest Ndukaife Anyabolu, Efosa Oviasu

**Affiliations:** ^1^Division of Nephrology/Rheumatology, Department of Internal Medicine, Federal Medical Centre, Umuahia, Abia State, Nigeria; ^2^Division of Rheumatology, Department of Internal Medicine, Lagos State University Teaching Hospital, Ikeja, Lagos State, Nigeria; ^3^Division of Ophthalmology, Federal Medical Centre, Umuahia, Abia State, Nigeria; ^4^Division of Nephrology, Department of Internal Medicine, Abia State University Teaching Hospital, Aba, Abia State, Nigeria; ^5^Division of Nephrology, Department of Internal Medicine, Chukwuemeka Odumegwu Ojukwu University Teaching Hospital, Awka, Nigeria; ^6^Division of Nephrology, University of Benin Teaching Hospital, Benin City, Nigeria

## Abstract

**Background and Objectives:**

The prevalence of noncommunicable diseases like chronic kidney disease is on the rise in third-world countries. In Nigeria and most sub-Saharan African countries, there is dearth of community-based studies on prevalence and predictors of chronic kidney disease, prompting us to undertake this study.

**Materials and Methods:**

This was a cross-sectional study, aimed at ascertaining the prevalence and predictors of chronic kidney disease (CKD) in a semiurban community in Lagos, Southwest Nigeria. The study's subjects were recruited from Agbowa community in Ikosi-Ejirin Local Council Development Area of Lagos state. The community was randomly selected. Questionnaires were used to obtain relevant information from the subjects. Body mass index, anthropometric measurements, and other relevant data were also collected.

**Results:**

CKD was observed in 30 subjects given prevalence of 7.5% in the community. Nine out of the 30 subjects (30%) with CKD were males, while 21 (70%) subjects were females. The prevalence of CKD was significantly higher in the female population. 28 of the subjects with CKD were in stage 3, while 2 of the subjects with CKD were in stage 4. Age, hypertension, and hyperuricemia were significantly associated with CKD. Using multiple logistic regression analysis, 4 variables predicted CKD in the study population. These were age (P =0.01, OR = 0. 274, CI = 0.102 – 0.739), hypertension (p = 0.011, OR = 0. 320, CI = 0.132 – 0.773), hyperuricemia (p=0.001, OR = 0.195, CI =0.083 – 0.461), and female sex (p = 0.009, OR = 3.775, CI = 1.401 – 10.17).

**Conclusion:**

The prevalence of CKD in the population is low compared with other studies from other parts of the country, and the predictors included age, hypertension, hyperuricemia, and female gender. This is the first community-based study in Nigeria to identify hyperuricemia as a risk factor for chronic kidney disease in the country.

## 1. Introduction

Chronic kidney disease (CKD) is defined by the Kidney Disease: Improving Global Outcome (KDIGO) as kidney damage that has continued for more than 3 months as characterized by structural or functional abnormalities of the kidney, with or without decreased glomerular filtration rate (GFR). It is also defined as GFR < 60mL/min/1.73 m^2^ for more than 3 months, with or without structural kidney damage. CKD is recognized as a risk factor for cardiovascular disease and is a major public health problem globally [1–3]. It is a disease condition associated with premature mortality, increased healthcare expenditures, and decreased quality of life. The terminal stage of the disease, End-Stage Renal Disease (ESRD), necessitates dialysis or kidney transplantation.

Studies have shown that the prevalence of CKD is rising in developing countries, like Nigeria, when compared with developed countries of the world due to double burden of communicable and non-communicable diseases in developing countries of the world [4]. There is dearth of population-based studies in Nigeria on prevalence of CKD, prompting us to undertake this study in a semiurban community in Lagos, Nigeria.

## 2. Methodology

The study was a community-based descriptive cross-sectional study. It was carried out in Agbowa, a community in Ikosi-Ejirin Local Council Development Area, Lagos, Nigeria. Agbowa is a semiurban community, located in the outskirt of metropolitan city of Lagos. It is located about 20 km from the major city of Lagos, with a projected population of 12,470 in 2016 based on the 2006 figure of 8,892 from the National Population Census carried out in Nigeria [5]. The community has equal gender distribution and 52% of the population are adults. The study was carried out from September 2016 to December 2016.

### 2.1. Study Design

The study was a community-based descriptive cross-sectional study.

### 2.2. Ethical Clearance

Ethical clearance was obtained for the study on prevalence of gout in a semiurban community in Lagos, and data for this study was obtained from the data bank.

#### 2.2.1. Sample Size Determination

The minimum sample size was determined using modified Fisher's formula:

(1) $$\begin{array}{c} n=\frac{Z2pq}{d2} \end{array}$$

where n is minimum sample size. p is estimated prevalence of the disease (12.3% from a previous study) = 0.123. q= 1-p = 1 – 0.123 = 0.877. d is tolerable sample error (0.05). Z=1.96 (standard normal deviation for 95% confidence interval).

Thus,

(2) n=1.96×1.96×0.123×0.8770.05×0.05n=165.8

Thus, a minimum of 165 subjects were to be recruited. However, to make allowance for 10% nonresponders and to increase the scope of the study, the sample size was increased to 400.

### 2.3. Study Population and Sampling Method

The study population was made up of 25% of adults aged 18 years and above living in the study area during the study period. Thus, a total of 1621 adults were interviewed and 400 subjects were recruited for the study. The inclusion criteria were adults ≥ 18 years who gave informed consent to the study. The exclusion criteria were subjects below 18 years and those who refused to give informed consent.

Systematic sampling technique was used. Ikosi-Ejirin Local Council Development Area (LCDA) was randomly selected by balloting from 37 LCDA in Lagos state. At Ikosi-Ejirin LCDA, the Agbowa community was randomly selected by balloting from the communities in the LCDA. The Agbowa community town hall, which is centrally located, was used as the study centre. Every fourth consenting adult interviewed was included in the study. This was arrived at by dividing the target study population of 1621 adults by the sample size of 400 subjects.

An advocacy visit was paid to the LCDA chairman for permission to carry out the study. Permission was also obtained from the traditional ruler of the community, ward leaders, and village heads. Awareness for the study was created through town criers as well as mosque and church announcements.

Ten research assistants were trained to assist in the study. These assistants were paramedics and nurses working in the community. They had good command of both English as well as Yoruba languages. The training included basic methods of taking anthropometric measurements, blood pressure check, and completion of questionnaires. The sampling technique and other protocols were emphasized. The researchers made sure that all assistants adhered to protocol. A researcher-administered questionnaire was completed per subject.

### 2.4. Anthropometry and Blood-Pressure Measurement

The blood pressure of the subjects was measured thrice after sitting for about 5 minutes. The three readings were taken at 2-minute intervals. The average reading for the last two was then recorded. A stadiometer was used to measure the height in centimeters. The weight of the subjects was measured in kilograms, using a weighing scale. The BMI was then calculated from the height and weight measured.

### 2.5. Laboratory Investigation

Investigations performed included serum electrolyte, urea and creatinine, fasting blood sugar, serum uric acid, and fasting lipid profile. The biochemical analysis was carried out in the chemical pathology laboratory of Lagos state's University Teaching Hospital Lagos, with the exception of the fasting blood sugar, which was carried out at the site using a glucometer. Estimated GFR was calculated from serum creatinine using the CKD-EPI collaboration equation.

### 2.6. Definition of Terms

#### 2.6.1. Hypertension

Subjects having systolic blood pressure of 140 mmHg and above or diastolic blood pressure of 90 mmHg and above or who had normal blood pressure but were pharmacologically treated for hypertension were categorized as hypertensive subjects [6, 7].

#### 2.6.2. Diabetes Mellitus

A history of previously known diabetes or a fasting plasma glucose of 126 mg/dL or more, and impaired fasting glucose defined as fasting plasma glucose of 100–125 mg/dL or a random blood glucose of 180 mg/dL or higher, was classified as diabetes mellitus, and impaired glucose tolerance was defined as random blood glucose between 140 and 180 mg/dL [8].

#### 2.6.3. Waist Circumference

WC was measured using a nonstretchable fiber measuring tape. The participants were asked to stand erect in a relaxed position with both feet together on a flat surface; one layer of clothing was allowed. It was measured to the nearest 0.5 cm at the high point of the iliac crest at minimal respiration [9].

#### 2.6.4. BMI

BMI was measured with the WHO classification [9] as follows: underweight BMI below 18.5 kg/m^2^, normal weight 18.5–24.9 kg/m^2^, and overweight BMI 25–29.9 kg/m^2^. BMI of 30–34.9 kg/m^2^ defines class I obesity, BMI of 35–39.9 kg/m^2^ defines class II obesity, and BMI of 40 kg/m2 and above was used to define class III obesity.

#### 2.6.5. Chronic Kidney Disease

This is defined as creatinine clearance or GFR <60 mL/min/1.73 m^2^ [1].

CKD-EPI collaboration creatinine equation [10] is

(3) GFR=141×min⁡Scrκ,1α×max⁡Scrκ,1−1.209×0.993Age×1.018  if  female×1.159  if  black

where were Scr is serum creatinine in mg/dL, *κ* is 0.7 for females and 0.9 for males, *α* is -0.329 for females and -0.411 for males, min indicates the minimum of Scr/*κ* or 1, and max indicates the maximum of Scr/*κ* or 1.

### 2.7. Data Analysis

Data obtained were entered using EpiData Software Version 3.1 (EpiData Association, Odense, Denmark), while analysis was carried out using SPSS Version 17.0 (SPSS Inc., Chicago, Illinois, USA). Relevant means and standard deviation were calculated for continuous variables. Findings were presented using relevant frequency tables and appropriate charts.  Bivariate logistic regression was used to determine factors associated with chronic kidney disease. Odds ratio (OR) and confidence intervals (CI) were determined. P value less than 0.05 was regarded as being statistically significant.

## 3. Results

### 3.1. Sociodemographic Characteristics of the Respondents

A total of 400 subjects were recruited for this study. There were 179 (44.75%) male and 221 (55.25%) female participants. The male : female ratio was 1:1.2. This was not statistically significant. The age range of the subjects was 18-88 years. Mean age was 46.5±16.0 years. Subjects in the age range of 36-45 years constituted majority of the study population (121 subjects, 30.25%), while those aged above 75 years constituted the minority (23 subjects, 5.75%). There was no statistically significant difference between the mean ages of male and female subjects (t=1.822, p=0.069). Majority of the participants were traders, artisans, and farmers, while 60 subjects (15%) were unemployed. Most of the subjects had formal education. See Table 1 for more details.

### 3.2. Blood Pressure, Blood Glucose, and Obesity in the Community

**Figure 1:**
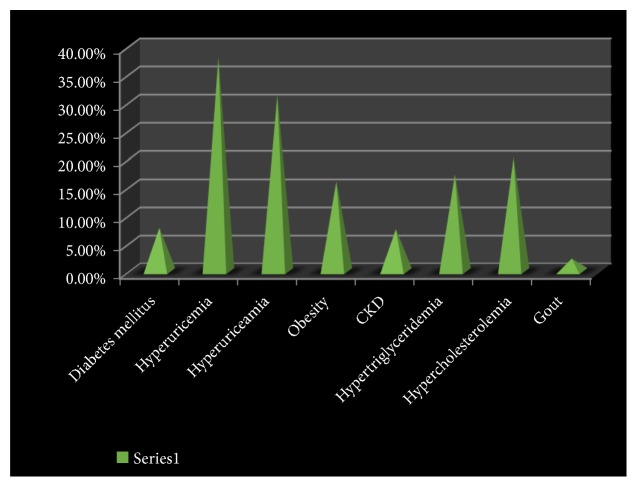
Prevalence of some medical condition in the community.

152 of the subjects (38%)} had hypertension, and 19 (7.75%) had diabetes mellitus. 64 subjects (16%) had obesity. See Figure 1.

### 3.3. Prevalence of CKD

**Figure 2:**
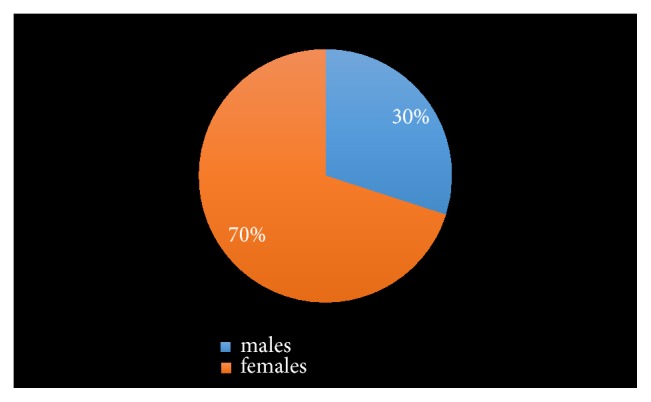
Relative prevalence of moderate-to-severe CKD among males and females.

**Figure 3:**
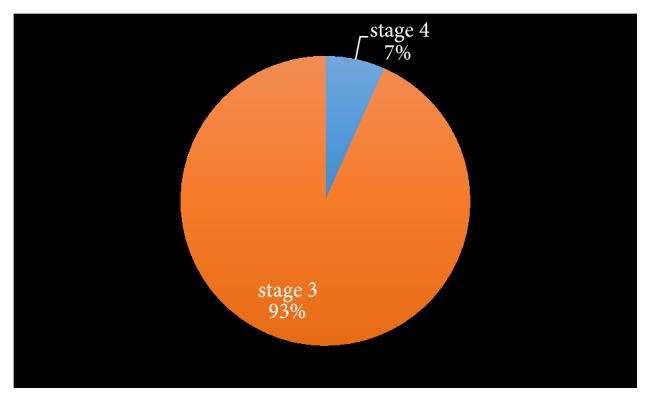
Relative prevalence of moderate-to-severe stages of CKD in the community.

The prevalence of CKD in the population is 7.5% (30 subjects). 9 (30%) of the subjects were males, while 21 (70%) subjects were females. The prevalence of CKD was significantly higher in the female population. 28 of the subjects with CKD were in stage 3, while 2 of the subjects with CKD were in stage 4. None of the subjects was in stage 5 (Figures 2 and 3).

### 3.4. Predictors of CKD

Bivariate logistic analysis was used to determine the relationship of CKD to other variables. Age, hypertension, and hyperuricemia were significantly associated with obesity (Table 2).

## 4. Discussion

CKD prevalence is noted to be on the increase in sub-Saharan African countries and other countries of the world. It is estimated that about 10% of the world population is affected by CKD [4]. There is increased cardiovascular mortality in subjects with CKD as well as loss of disability-adjusted life years. In low- and middle-income countries of the world, there is absence of kidney registries, making it difficult to ascertain the true burden of CKD. The increase in prevalence of diabetes mellitus, obesity, aging, and hypertension is attributed to be responsible for the increase in prevalence of CKD globally. A good majority of the subjects that receive treatment for CKD are in the developed countries of the world. There is a high mortality rate in subjects with CKD in low- and middle-income countries due to the fact that they cannot afford the cost of renal replacement therapy due to lack of access to universal healthcare and prevailing poverty [11, 12].

The prevalence of CKD in the semiurban community was 7.5%. The males constituted 30% of the subjects, while females constituted 70%. The prevalence of CKD observed in this study is lower than the prevalence observed in a previous community-based study that also used the CKD-EPI creatinine equation in estimating the prevalence of CKD in another location in Nigeria [13]. The prevalence of CKD observed in the study was 11.4%. The lower prevalence observed in this study may be due to the fact that the number of subjects that were recruited for the study was lower (400 subjects) than the number of subjects that took part in the latter other study (1941).

The prevalence of CKD observed is also much lower than that obtained in 2 community-based studies on CKD which used the Cockcroft–Gault (CG) formula in estimating the prevalence of CKD [14, 15]. This may be explained by the fact that the CG formula has been shown to overestimate moderate-to-severe stages of CKD and is less reliable in estimating the glomerular filtration rate when compared to the CKD-EPI creatinine equation [4].

The prevalence is also lower when compared with previous community-based studies on CKD [16–18] in 3 other locations in Nigeria, which used the 4-parameter MDRD equation in estimating the prevalence of CKD. The CKD-EPI creatinine equation is slightly better than the MDRD equation in estimating the GFR; however, this reason may not be enough for the differences observed. The difference in dietary habits, due to difference in ethnicity, may also be a contributory factor.

The prevalence of CKD is much higher in the female population when compared with the males. This finding is in keeping with other population-based studies on prevalence of CKD in other locations in Nigeria, which showed that the prevalence of CKD is higher in females [4]. Similar observations were also made from several other studies [4, 19, 20]. The difference in glomerular structure and hormone metabolism and the fact that females have less muscle mass than males are believed to play a major role in the differences in prevalence of CKD between female and male gender[10, 21].

Using multiple logistic regression analysis, 4 variables predicted CKD in the study population. These were age, hypertension, hyperuricemia, and female gender. Age and hypertension had been identified previously as risk factors of CKD in community-based studies on prevalence of CKD in Nigeria [14, 18]. Female gender was identified as risk factor for CKD in one of the studies [14]. None of the earlier community-based studies in Nigeria identified hyperuricemia as risk factor. However, a systematic review and meta-analysis based on observational cohort studies involving 13 studies carried out in other parts of the globe showed that hyperuricemia is a risk factor for CKD [22].

## 5. Conclusion

The study showed that the prevalence of CKD in Ikosi-Ejirin LCDA was 7. 5%. This is lower than the other results obtained from earlier community-based studies carried out in Nigeria. The prevalence of CKD was higher in the female gender as previously observed in earlier community-based studies. The study also showed that CKD is associated with age, hypertension, hyperuricemia, and female gender.

The preventive strategies to stem the tide of CKD should involve identifying those at risk of developing CKD; educating the population on how to prevent kidney disease; promoting the awareness among healthcare providers, general public, local government, state government, and federal government policy-makers on health; modifying lifestyle suitable for susceptible individuals; and providing needed facilities in primary, secondary, and tertiary hospitals in the country for detecting early stage of CKD [23].

## 6. Limitations

The sample size for this study was small, and also the estimation of GFR using CKD-EPI creatinine equation should have been repeated 3 months later in keeping with the K/DOQI practice guidelines.

## Figures and Tables

**Table 1:** Sociodemographic characteristics of the subjects.

Characteristics		Number (N)	Percentage (%)
Sex	Male	179	44.7
	Female	221	55.3

Age range (years)	≤25	39	9.7
	26 – 35	50	12.5
	36 – 45	121	30.2
	46 – 55	81	20.3
	56 – 65	48	12.0
	66 – 75	38	9.5
	>75	23	5.8

Marital status	Single	75	18.8
	Married	297	74.2
	Divorced/Separated	6	1.5
	Widow/widower	22	5.5

Religion	Islam	164	41.0
	Christianity	228	57.0
	Others	8	2.0

Postmenopausal (n=221)	Yes	73	33.0
	No	148	67.0

Education	No formal education	65	16.3
	Primary level	121	30.2
	Secondary level	177	44.3
	Tertiary level	37	9.2

Occupation	Trader	126	31.5
	Artisan	102	25.5
	Clerical officers	6	1.5
	Professionals	22	5.5
	Clergy	8	2.0
	Farming	65	16.2
	Unemployed	60	15.0
	Retired	9	2.3
	Student	2	0.5

**Table 2:** Bivariate analysis of factors associated with CKD.

Variable	*p*-value	Odds ratio	Confidence interval
Age >45 years	0.01	0.274	0.102 – 0.739
Hypertension	0.011	3.320	0.132 – 0.773
Hyperuricemia	0.001	0.195	0.083 – 0.461
Female sex	0.009	3.775	1.401 – 10.17

## Data Availability

The data for this paper are always available whenever needed.

## References

[1] Levey A. S., Eckardt K. U., Tsukamoto Y. (2005). Definition and classification of chronic kidney disease: a position statement from Kidney Disease: improving Global Outcomes (KDIGO). *Kidney International*.

[2] National Kidney Foundation (2002). K/DOQI Clinical practice guidelines for chronic kidney disease: evaluation, classification and stratification. *American Journal of Kidney Diseases*.

[3] Sarnak M. J., Levey A. S., Schoolwerth A. C. (2003). Kidney disease as a risk factor for development of cardiovascular disease: a statement from the American Heart Association Councils on kidney in cardiovascular disease, high blood pressure research, clinical cardiology, and epidemiology and prevention. *Hypertension*.

[4] Chukwuonye I. I., Ogah O., Anyabolu E. (2018). Prevalence of chronic kidney disease in Nigeria: systematic review of population-based studies. *International Journal of Nephrology and Renovascular Disease*.

[5] Federal Republic of Nigeria (2009). *Legal Notice on Publication of 2006 Census Final Results, Official Gazette*.

[6] Hansson L., Hedner T., Himmelmann A. (1999). The 1999 WHO-ISH Guidelines for the Management of Hypertension – new targets, new treatment and a comprehensive approach to total cardiovascular risk reduction. *Blood Pressure, Supplement*.

[7] Chobanian A. V., Bakris G. L., Black H. R. (2003). Seventh report of the joint national committee on prevention, detection, evaluation, and treatment of high blood pressure. *Hypertension*.

[8] World Health Organization (1999). *Definition, Diagnosis and Classification of Diabetes Mellitus and Its Complications*.

[9] Chukwuonye I. I., Chuku A., Onyeonoro U. U. (2013). Prevalence of abdominal obesity in Abia State, Nigeria: results of a population-based house to house survey. *Diabetes, Metabolic Syndrome and Obesity: Targets and Therapy*.

[10] Levey A. S., Stevens L. A., Schmid C. H. (2009). A new equation to estimate glomerular filtration rate. *Annals of Internal Medicine*.

[11] Perazella M. A., Khan S. (2006). Increased mortality in chronic kidney disease: a call to action. *The American Journal of the Medical Sciences*.

[12] Couser W. G., Remuzzi G., Mendis S., Tonelli M. (2011). The contribution of chronic kidney disease to the global burden of major noncommunicable diseases. *Kidney International*.

[13] Ulasi I. I., Ijoma C. K., Onodugo O. D., Arodiwe E. B., Ifebunandu N. A., Okoye J. U. (2013). Towards prevention of chronic kidney disease in Nigeria: a community-based study in Southeast Nigeria. *Kidney International Supplements*.

[14] Okoye O., Okoye A., Oviasu E., Ojogwu L. (2011). Prevalence of chronic kidney disease and its risk factors amongst adults in a rural population in Edo State, Nigeria. *Journal of US-China Medical Science*.

[15] Nalado A., Abdu A., Adanu B. (2016). Prevalence of chronic kidney disease markers in Kumbotso rural Northen Nigeria. *African Journal of Medical and Health Sciences*.

[16] Oluyombo R., Ayodele O. E., Akinwusi P. O. (2013). A community study of the prevalence, risk factors and pattern of chronic kidney disease in Osun State, South West Nigeria. *West African Journal of Medicine*.

[17] Oluyombo R., Olamoyegun M. A., Ayodele O. E., Akinwusi P. O., Akinsola A. (2017). Clustering of chronic kidney disease and cardiovascular risk factors in South-West Nigeria. *Journal of Nephropathology*.

[18] Okwuonu C., Chukwuonye I., Adejumo O., Agaba E., Ojogwu L. (2017). Prevalence of chronic kidney disease and its risk factors among adults in a semi-urban community of South-East Nigeria. *Nigerian Postgraduate Medical Journal*.

[19] Zhang Q., Rothenbacher D. (2008). Prevalence of chronic kidney disease in population-based studies: systematic review. *BMC Public Health*.

[20] Kalyesubula R., Nankabirwa J. I., Ssinabulya I. (2017). Kidney disease in Uganda: a community-based study. *BMC Nephrology*.

[21] Hecking M., Bieber B. A., Ethier J. (2014). Sex specific differences in hemodialysis prevalence and practices and the male to – female mortalityrate: the dialysis outcomes and practice patterns study (DOPPS). *PLoS Medicine*.

[22] Li L., Yang C., Zhao Y., Zeng X., Liu F., Fu P. (2014). Is hyperuricemia an independent risk factor for new-onset chronic kidney disease?: a systematic review and meta-analysis based on observational cohort studies. *BMC Nephrology*.

[23] Ayodele O. E., Alebiosu C. O. (2010). Burden of chronic kidney disease: an international perspective. *Advances in Chronic Kidney Disease*.

